# Emerging Treatments for Advanced/Metastatic Pheochromocytoma and Paraganglioma

**DOI:** 10.1007/s11864-020-00787-z

**Published:** 2020-08-29

**Authors:** Maran Ilanchezhian, Abhishek Jha, Karel Pacak, Jaydira Del Rivero

**Affiliations:** 1 Pediatric Oncology Branch, Center for Cancer Research, National Cancer Institute, Bethesda, MD USA; 2 Eunice Kennedy Shriver National Institute of Child Health and Human Development, National Institutes of Health, Bethesda, MD USA; 3 Developmental Therapeutics Branch, Rare Tumor Initiative, Center for Cancer Research, National Cancer Institute, Bethesda, MD USA; 4 Medical Oncology & Clinical Endocrinology, Center for Cancer Research, National Cancer Institute/National Institutes of Health, 10 Center Drive, MSC 1906, Building 10, CRC 13C-434, Bethesda, MD 20892 USA

**Keywords:** Pheochromocytoma, Paraganglioma, Genetics, Imaging, Oncogenic signaling, Therapy

## Abstract

The incidence of metastatic pheochromocytoma (PHEO) and paraganglioma (PGL) may occur in as many as 35% of patients particularly with PGL and even more frequently in those with specific mutations. Biochemical, morphological, and molecular markers have been investigated for use in the distinction of benign from malignant PHEO/PGL. PHEO/PGL metastasizes via hematogenous or lymphatic routes and shows differences based on mutational status. The most common sites of involvement in patients that have an SDHB mutation are the bone (78%), lungs (45%), lymph nodes (36%), and liver (35%). In patients with sporadic PHEO/PGL, the most common sites of metastasis are the bones (64%), lungs (47%), lymph nodes (36%), and liver (32%). Metastases may be present at presentation or may occur later. Metastases to the liver and lungs are associated with a shorter survival. Overall, the estimated 5-year survival rates are between 34 and 74%. Currently, treatments for metastatic PHEO/PGL are essentially palliative. Surgery is potentially curative; however, tumor dissemination limits the chance for a curative resection. When surgical intervention is not amenable, the therapeutic options include radiolabeled MIBG (Azedra®—iobenguane 131 was recently FDA-approved for patients > 12 years and older with iobenguane scan positive) or systemic chemotherapy with cyclophosphamide, vincristine, and dacarbazine (CVD) with an overall objective response rate (ORR) of less than 40%; however, it is not clear if the administration of CVD impacts overall survival, as nearly all patients develop progressive and ultimately fatal disease. Other treatment modalities under investigation include cytoreductive techniques, novel radiopharmaceuticals, chemotherapy, radiotherapy, immunotherapy, and experimental therapies. Here we are discussing emerging treatment for advanced/metastatic PHEO/PGL.

## Introduction

Pheochromocytomas (PHEOs) and paragangliomas (PGLs) are rare neuroendocrine tumors that arise from chromaffin cells. PHEOs arise from the adrenal medulla, while PGLs arise from the neural crest progenitors located outside of the adrenal gland [1]. The incidence of PHEO/PGL is estimated to be around 1 per 300,000 people, with an average diagnosis age of 40 [2, 3]. The clinical presentation of PHEO/PGL is variable and overlaps with similar symptoms occurring in other disease conditions. Most of the symptoms of PHEO/PGL are an effect of the overproduction of catecholamines [4]. These include hypertension, headache, palpitations, and anxiety. Hypertension is the most common presenting symptom and can be paroxysmal or sustained. Some patients may present with orthostatic hypotension [5]. Sympathetic PHEO/PGLs frequently produce considerable amounts of catecholamines and are found in the adrenal medulla in 80% of patients [1, 6]. There are no curative treatments for metastatic PHEO/PGL and current systemic therapies are accompanied with side effects that can interfere with the quality of life. In patients with metastatic PHEO/PGL with no evidence of disease progression and asymptomatic, active surveillance is appropriate [7, 8]. If evidence of disease progression and symptomatic, multiple lines of treatment are considered, including hormonal symptoms control, surgery of the primary tumor and/or metastases, ablation/embolization, radiotherapy, and systemic therapies [7, 9, 10] in metastatic PHEO/PGL. Moreover, excessive hormone production (epinephrine and/or norepinephrine) is complicated with cardiovascular disease and gastrointestinal dysfunction [11, 12].

## Genetics

**Fig. 1:**
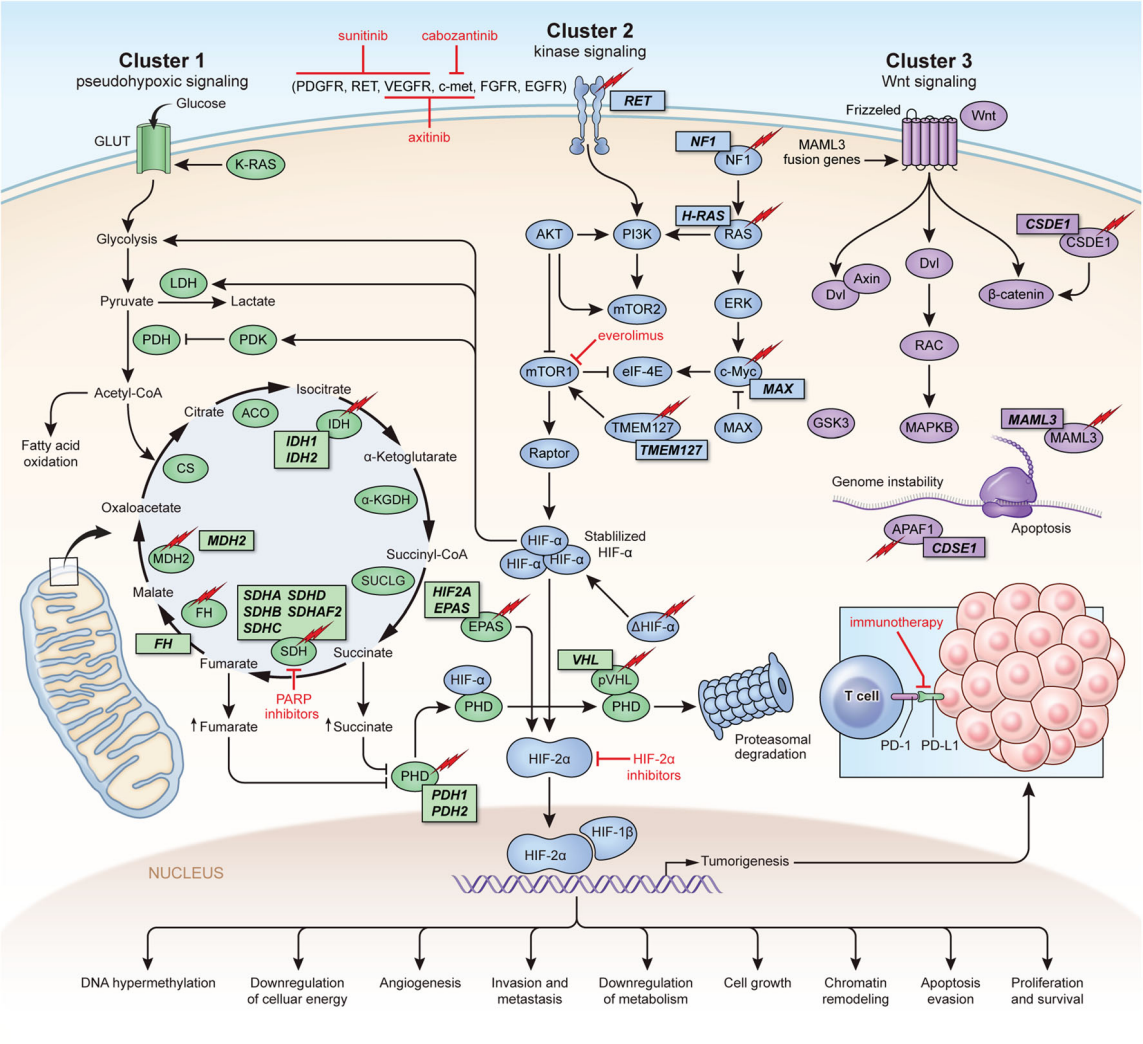
Genetics and molecular pathways for pheochromocytoma and paragangliomas. Clusters I, II, and III with molecular-targeted options. Cluster I PHEOs/PGLs, also known as the pseudohypoxia group, are characterized by mutations in *SDHA*, *SDHB*, *SDHC*, *SDHD*, *SDHAF2*, *FH*, *VHL*, and EPAS1 [20]. Cluster II PHEOs/PGLs, also known as the kinase signaling group, are characterized by mutations in *RET*, *NF1*, *TMEM127*, *MAX*, and *HRAS* [16]. The Wnt signaling group (cluster III) includes mutations in the genes *CSDE1* and *MAM* [73, 74].

The majority of PHEOs/PGLs are sporadic tumors and of these, 25–30% are associated with somatic mutations [13]. About 35% are familial in origin where patients are found to harbor germline mutations in over 20 susceptibility genes [14, 15]. The mutations associated with PHEO/PGL can be grouped into 3 clinically relevant clusters: pseudohypoxia, kinase signaling, and Wnt signaling [16•, 17••]. The pseudohypoxia group (cluster I) contains mutations in *SDHA*, *SDHB*, *SDHC*, *SDHD*, *SDHAF2*, *FH*, *VHL IDH1/2*, *MHD2*, *PHD1/2*, and *HIF2/EPAS1* [18, 19••]. This group exhibits metabolic reprogramming and pseudohypoxic signaling that are hallmarks of the aforementioned mutations [20]. The kinase signaling group (cluster II) consists of mutations in *RET*, *NF1*, *TMEM127*, *MAX*, and *HRAS* [16•]. The Wnt signaling group (cluster III) includes *CSDE1* and *MAML3* gene mutations. Patients with mutations in this group exclusively present as somatic mutations and it has been proposed that this group of mutations results in more aggressive PHEOs/PGLs [16•, 21, 19••] (Fig. 1).

Succinate dehydrogenase (*SDH*) mutations are found in approximately 27% of patients with advanced PHEO/PGL [22]. Astuti et al. first described in 2001 that mutations in succinate dehydrogenase subunit B (*SDHB)* have been linked to more aggressive tumor behavior, demonstrated as a higher rate of metastasis [23–26••]. The rate of metastasis of *SDHB*-related PHEOs/PGLs has been reported to be between 34% [22] and 71% [27], with a 5-year survival rate of 36% after the diagnosis of metastasis [28]. The metastatic potential attributed to mutations in the other SDH subunits has been described as 21% in *SDHA*, rarely malignant in *SDHC*, and < 10% in *SDHD* [29].

## Diagnosis of PHEO/PGL

**Fig. 2:**
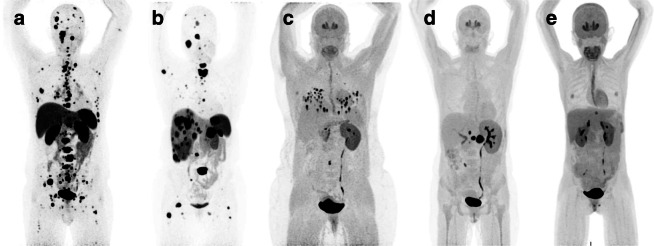
This figure demonstrates the preferred radionuclide to be used in various cohorts of pheochromocytoma (PHEO) and/or paraganglioma (PGL) per latest joint guidelines proposed by European Association of Nuclear Medicine and Society of Nuclear Medicine and Molecular Imaging. The preferred radionuclide in metastatic *SDHB-*related PHEO/PGL (**a**) and metastatic sporadic-PHEO/PGL (**b**) is ^68^Ga-DOTATATE. However, ^18^F-FDOPA is the radionuclide of choice in metastatic *VHL*- (**c**), *HIF2A*- (**d**), and *RET*- (**e**) mutated patients.

The diagnosis of PHEO and PGL is based on the presence of symptoms, biochemical confirmation, and different imaging modalities. Biochemical testing is based on the continuous production of catecholamines and their metabolites are called metanephrines [6, 30]. Imaging procedures include anatomical and functional techniques [31, 32]. Ga-68 DOTATATE and other diverse radionuclide imaging techniques are available for the diagnosis, staging, and follow-up of PHEO/PGL and an attempt has further been made to characterize the radionuclide of choice across the genotypes [31, 32] (Fig. 2).

## Management of advanced disease

The frequency of metastatic PHEOs/PGLs in certain genetic disorders ranges from 1 to 90%. *SDH* mutations are found in approximately 30% of patients with metastatic PHEO/PGL [33]. *SDHB* mutations have been linked to more aggressive tumor behavior, and are more likely to present with metastatic disease than patients with sporadic PHEOs/PGLs [23–25]. Eisenhofer et al. have described an increase in the likelihood of metastases in PHEOs from less than 6% for tumors smaller than 5 cm to over 50% in tumors larger than 10 cm; for PGLs, the rate of malignancy increases to over 80% for tumors larger than 9 cm [34].

*SDHB*-related PHEO/PGL, extra-adrenal location, younger age at initial presentation, larger size of the primary tumor, and elevated norepinephrine, dopamine, and its metabolite methoxytyramine levels have been explored as risk factors for the metastatic behavior of PHEOs/PGLs [23, 35–38]. While histopathological characteristics of tumors may not show a definite diagnosis of malignancy, clinical correlates, such as a tumor weight > 80 g, high tumor concentration of dopamine, tumor size > 5 cm, presence of confluent tumor necrosis, extra-adrenal tumor location, adrenal PHEO that does not take up metaiodobenzylguanidine (iobenguane, MIBG), and a younger age, have been associated with an increased likelihood for metastatic behavior [39].

**Fig. 3:**
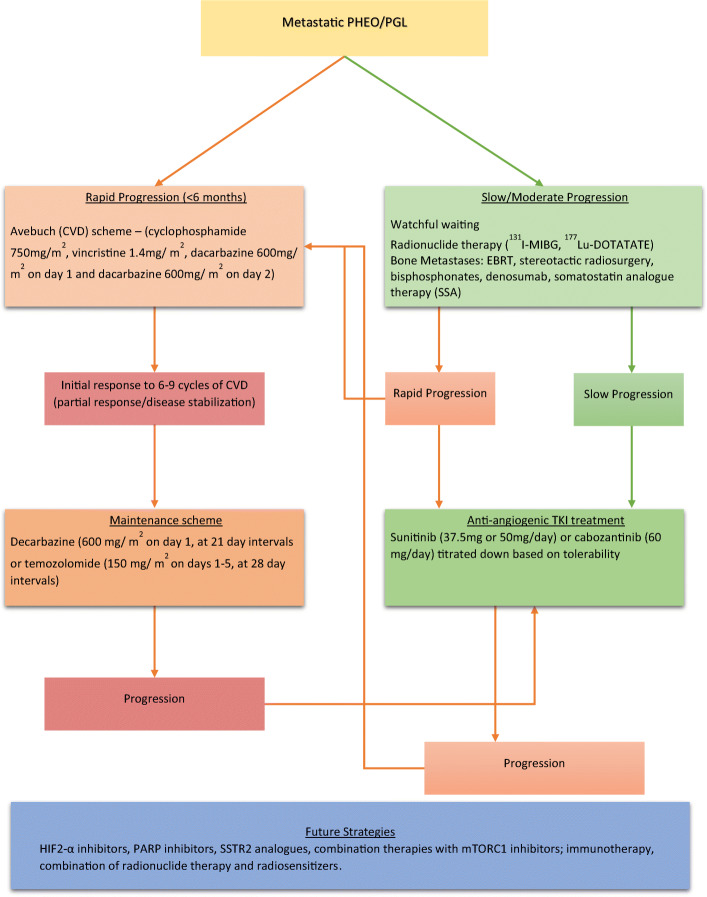
Treatment algorithm for metastatic PHEO/PGL. PHEO, pheochromocytoma; PGL, paraganglioma; CVD, cyclophosphamide, vincristine, dacarbazine; I-MIBG, iodine metaiodobenzylguanidine; Lu-DOTATATE, Lutathera-DOTATATE; cEBRT, conventional external beam radiation therapy; HIF2-α, hypoxia-inducible factor 2-alpha; PARP, poly (ADP-ribose) polymerase; SSTR2, somatostatin receptor 2; mTORC1, mammalian target of rapamycin complex 1. Modified from Nolting et al. (Cancers 2019) [18].

At present, most treatments for metastatic PHEOs/PGLs are palliative. Surgery is potentially curative; however, tumor dissemination limits the chance for a curative resection [40]. Other treatment modalities include cytoreductive techniques, radiopharmaceuticals, chemotherapy, radiotherapy, and experimental therapies. Targeted radiotherapy using ^131^I MIBG (Azedra®) is an option in systemic treatment. Radiolabeled somatostatin analogues are being investigated [41, 42]. Less than 40% of patients with metastatic PHEO respond (usually partial rather than complete response) to currently used therapeutic modalities such as ^131^I MIBG or chemotherapy [43] (Fig. 3).

## Pseudohypoxia group (cluster I) targeted therapies

Cluster I PHEOs/PGLs, also known as the pseudohypoxia group, are characterized by mutations in *SDHA*, *SDHB*, *SDHC*, *SDHD*, *SDHAF2*, *FH*, *VHL*, and EPAS1 [20].

### Antiangiogenic therapy

PHEOs/PGLs are highly vascularized tumors, which support the notion that anti-angiogenic therapies may be options for treatment [44, 45]. Vascular endothelial growth factor (VEGF), a well characterized angiogenic factor, has been shown to be upregulated in metastatic PHEO, which suggests that it could be a potential therapeutic target [46]. Currently, human VEGF-A monoclonal antibodies and tyrosine kinase inhibitors are used as angiogenic therapies. These therapies have already been approved for patients with advanced cell renal carcinoma, including patients with *SDHB* mutations. Several case reports have also shown a partial response or stable disease in cluster I PHEOs/PGLs treated with tyrosine kinase inhibitors [47–50]. Ongoing trials are being conducted to explore the efficacy of tyrosine kinase inhibitors in advanced/metastatic PHEO/PGL (Table 1). Two phase II trials are currently under way to study the response of patients with metastatic PHEO/PGL to sunitinib. One of these trials (NCT00843037) has shown a disease control rate of 83% (3/25 patients achieved a partial response and 16/25 patients had stable disease). All patients who responded were carriers of germline mutations in the cluster I genes, *SDHA*, *SDHB*, or in *RET* [51]*.* In addition, two phase II trials for the tyrosine kinase inhibitor axitinib are also under way. Preliminary results from the NCT01967576 phase II trial of axitinib have shown partial responses in 3/9 patients as well as stable disease in 5 additional patients [52]. Phase II trials studying VEGF inhibitors, lenvatinib and dovitinib, are also in progress and both are enrolling patients with PHEO/PGL (Table 1, Fig. 1).

**Table 1:** Ongoing clinical trials for pheochromocytoma/paraganglioma patients

Therapy	Classification	Study design	Clinical trial number
Cluster I therapies			
Axitinib	TKI	Phase 2	NCT03839498
Axitinib	TKI	Phase 2	NCT01967576
Sunitinib	TKI	Phase 2	NCT01371201
Sunitinib	TKI	Phase 2	NCT00843037
Cabozantinib S-malate	TKI	Phase 2	NCT02302833
Lenvatinib	TKI	Phase 2	NCT03008369
Dovitinib	TKI	Phase 2	NCT01635907
Pembrolizumab	Immunotherapy	Phase 2	NCT02721732
Nivolumab, ipilimumab	Immunotherapy	Phase 2	NCT02834013
Other therapies			
131-I MIBG	Radioiodine	N/A	NCT01850888
131-I MIBG	Radioiodine	Phase 1	NCT03649438
Iobenguane I-131	Radioiodine	Phase 2	NCT00874614
Iobenguane I-131	Radioiodine	Phase 2	NCT00107289
Lanreotide	Somatostatin analogue	Phase 2	NCT03946527
Lu-177-DOTATATE	PRRT	Phase 2	NCT03206060
Lu-177 DOTATOC	PRRT	Phase 2	NCT04276597
Lu-177-DOTA-OCTREOTATE	PRRT	Phase 1/2	NCT03923257
EO2401 Vaccine	Microbial-derived peptide vaccine	Phase 1/2	NCT04187404
ONC201	DRD2 antagonist	Phase 2	NCT03034200
Tipifarnib	RAS inhibitor	Phase 2	NCT04284774

*TKI*, tyrosine kinase inhibitor; *PRRT*, peptide receptor radionucleotide therapy; *DRD2*, dopamine receptor D2

### Hypoxia-inducible factor (HIF) inhibitors

Abnormal activation of hypoxia signaling is another one of the characteristics of cluster I PHEOs/PGLs [53]. As a result, HIF inhibitors could be a potential therapy for patients with cluster I PHEO/PGL. Molecular studies have identified HIF-2α as one of the main oncogenic drivers of PHEOs/PGLs [44, 54–56]. PT2339 and PT2385 are two selective HIF-2α antagonists, which were developed and evaluated for their anti-tumor effects. In cell lines derived from VHL-mutated clear cell renal cell carcinomas (ccRCCs), PT2399 demonstrated a stronger suppression effect than sunitinib [57]. In a phase I clinical trial, PT2385 showed a complete response, partial response, and stable disease in 2%, 12%, and 52% of ccRCC patients, respectively [58]. A phase II clinical trial is currently ongoing to evaluate PT2385 in patients with VHL-mutated ccRCCs. While neither of these compounds has been used in patients with PHEO/PGL, the tumor-suppressing effects of HIF-2α inhibitors in HIF-driven tumors such as ccRCCs are promising and indicate that they could have potential therapeutic options for patients with cluster I PHEO/PGL. Furthermore, cluster I mutations including *SDHB*, *SDHD*, *VHL*, and *FH* have been associated with renal cell carcinomas, so agents that have shown efficacy with ccRCCs may warrant further study in PHEO/PGL patients [59]. Preclinically, anthracycleines (daunorubicin, doxorubicin, epirubicin, and idarubicin) have shown suppression of cell growth in metastatic PHEOs/PGLs through the inhibition of HIF-1 and HIF-2α and as such could be a new therapeutic option for patients with metastatic PHEO/PGL [60] (Fig. 1).

### Immunotherapy

Psuedohypoxia may prevent immune recognition of cluster I PHEOs/PGLs through the inactivation of cytotoxic T cell lymphocytes, activation of immune-suppressive monocytes (M2 macrophages), and increased expression of the immune checkpoint protein programmed death-ligand 1 (PD-L1) resulting in exhaustion of tumor-infiltrating lymphocytes [61–63]. For these reasons, specific immune system modulating approaches are being introduced to boost the immune microenvironment in these tumors followed by the use of current immunotherapies. Two phase II clinical trials of checkpoint inhibitors (nivolumab + ipilimumab, and pembrolizumab) are currently active that include PHEOs/PGLs (Table 1) and several new approaches are being studied experimentally [64] (Fig. 1).

### Poly (ADP-ribose) polymerase (PARP) inhibition

Germline loss-of-function mutations in succinate dehydrogenase, a key Krebs cycle enzyme, are linked to elevated levels of succinate [65, 66]. High levels of succinate and NAD^+^ inhibit homologous recombination (HR)-based DNA repair [67]. Poly (ADP-ribose) polymerase (PARP) is activated after DNA damage and regulates base excision repair, homologous recombination, and non-homologous end joining [68]. Therefore, *SDH*-mutated cluster I PHEOs/PGLs may be sensitive to treatment with these PARP inhibitors. Olaparib, an FDA-approved PARP inhibitor, was able to amplify the therapeutic effect of temozolomide in *SDHB-*mutant preclinical models [69] (Fig. 1).

## Kinase signaling group (cluster II) targeted therapies

Cluster II PHEOs/PGLs, also known as the kinase signaling group, are characterized by mutations in *RET*, *NF1*, *TMEM127*, *MAX*, and *HRAS* [16•].

### Mammalian target of rapamycin (mTOR) inhibition

Hyperactivation of kinase activity is commonly detected in the Ras/Raf/Erk or PI3K/Akt/mTOR pathways of patients with cluster II PHEO/PGL and mutations in *RET*, *NF1*, *TMEM127*, and *MAX* [70]. For this reason, kinase signaling inhibitors have been proposed for targeted therapeutics. Treatment using everolimus, an mTOR1 inhibitor, has been evaluated in patients with advanced/metastatic PHEO/PGL [71]. In a phase II study, 5 out of 7 patients with PHEO/PGL achieved stable disease on this therapy. The median progression-free survival was 3.8 months and the median duration of treatment was also 3.8 months for these patients [72].

## Wnt signaling group (cluster III) targeted therapies

The Wnt signaling group (cluster III) includes mutations in the genes *CSDE1* and *MAM.* Wnt signaling is involved not only in tumorigenesis and tumor proliferation but also in many essential physiological processes as well [73, 74]. As a result, there are no Wnt signaling-targeted therapies for PHEO/PGL patients. However, there are still many therapies that are not specific to any PHEO/PGL cluster that have shown promise for the treatment of PHEO/PGL. These therapies are described below.

## Other therapies

### ^131^I-MIBG

Iodine-131 metaiodobenzylguanidine (^131^I-MIBG), a radiopharmaceutical agent used for scintigraphic localization of PHEO/PGL, has been employed to treat metastatic PHEO/PGL since 1983. In a retrospective study, a total of 116 patients were evaluated. The majority of the patients were selected for treatment based upon positive tracer uptake studies. The cumulative dose of ^131^I-MIBG administered ranged from 96 to 2322 mCi (3.6 to 85.9 GBq), with a mean (± SD) of 490 ± 350 mCi (18.1 ± 13.0 GBq). The subjects received a mean single therapy dose of 158 mCi (5.8 GBq) and the number of doses administered ranged from 1 to 11, with a mean of 3.3 ± 2.2 doses. Initial symptomatic improvement was achieved in 76% of patients, tumor responses (partial or complete response) in 30%, and hormonal responses in 45%. Five patients had complete tumor and hormonal responses, ranging from 16 to 58 months. Patients with metastases to soft tissue had more favorable responses to treatment than those with metastases to bone. No difference was noted in the ages when responders were compared with non-responders. Adverse effects, recorded in 41% of the treated patients, were generally mild except for one fatality from bone marrow aplasia. Among 89 patients with follow-up data, 45% of the responders had relapsed with recurrent or progressive disease after a mean interval of 29.3 ± 31.1 months (median 19 months). Of patients with an initial response to ^131^I-MIBG, death was reported in 33% after a mean of 23.2 ± 8.1 months (median 22 months) following treatment. Of non-responders, death was reported in 45% after a mean of 14.3 ± 8.3 months (median 13 months) and it was concluded that ^131^I-MIBG therapy might be a useful palliative treatment [39, 75].

A meta-analysis by van Hulsteijn et al. reviewed seventeen studies encompassing a total of 243 patients with malignant PHEO/PGL who were treated with ^131^I-MIBG therapy. The analysis showed that stable disease could be seen in 52% of patients and a partial hormonal response in 40%. The 5-year survival rates reported were between 45 and 64%. The mean progression-free survival based on two studies in the analysis was 23 months and 28 months, respectively [76].

Recently, the FDA through fast tract designation approved Azedra® (a high-specific-activity ^131^I-metaiodobenzylguanidine (^131^I-MIBG) agent made of labeled ^131^I-MIBG molecules, allowing for lower mass doses of ^131^I-MIBG to be administered) for adult and pediatric patients (> 12 years old) with advanced, unresectable disease. The FDA approval was based on the results of a phase 2 open-label, multicenter trial that included 68 patients with pheochromocytomas or paragangliomas. The primary end point was a > 50% reduction of all antihypertensive medications lasting for at least 6 months. Twenty-five percent evaluable patients experienced a 50% or greater reduction of all antihypertensive medication for at least 6 months. Overall tumor response was achieved in 22% patients, and of those patients, 53% experienced durable tumor responses lasting 6 months or longer. These results suggest that ^131^I-MIBG can have clinical benefit in patients with locally advanced or metastatic PHEO/PGL [77, 76, 78].

### Chemotherapy

Combined chemotherapy with cyclophosphamide, vincristine, and dacarbazine (CVD) has emerged as a standard option [79]. Kaiser et al. first documented that in a case series of three patients receiving CVD, a marked decrease in blood pressure and an improvement in performance status were achieved within the first few cycles of treatment. At a follow-up of 6 to 13 months, all three patients continued to receive chemotherapy, with further regression of tumor in two and stable disease in one. CVD was well tolerated; moderate reversible granulocytopenia, neurotoxicity, and one episode of pneumonitis were the major toxicities encountered. This report suggested that combination chemotherapy appears to be effective for symptomatic malignant pheochromocytoma [80•]. Results of a non-randomized, single-arm trial including 14 patients with confirmed metastatic PHEO/PGL and elevated urinary catecholamine secretion have been reported. After optimization of antihypertensive therapy, patients received cyclophosphamide, 750 mg/m^2^ on day 1; vincristine, 1.4 mg/m^2^ on day 1; and dacarbazine, 600 mg/m^2^ on days 1 and 2, every 21 days. Combination chemotherapy with CVD produced a complete plus partial response rate of 57% (median duration, 21 months; range, 7 to more than 34). Complete and partial biochemical responses were seen in 79% of patients (median duration, more than 22 months; range, 6 to more than 35). All responding patients had objective improvement in performance status and blood pressure [81].

A long-term follow-up study was conducted in 18 patients treated with CVD at the National Institutes of Health. Combination chemotherapy with CVD produced a complete response rate of 11% and a partial response rate of 44%. Median survival was 3.8 years for patients whose tumors responded to therapy and 1.8 years for patients whose tumors did not respond (*p* = 0.65). All patients with tumors scored as responding reported improvement in their symptoms related to excessive catecholamine release and all had objective improvements in blood pressure. In this 22-year follow-up, there was no difference in overall survival between patients whose tumors objectively shrank and those with stable or progressive disease. However, patients reported improvement in symptoms had objective improvements in blood pressure and had tumor shrinkage that made surgical resection possible. The authors conclude that CVD therapy is not indicated in every patient with metastatic PHEOs/PGLs, but should be considered in the management of patients with symptoms and where tumor shrinkage might be beneficial [82].

A retrospective review of patients treated with CVD included 17 cases. The follow-up period after initiation of CVD ranged from 12 to 192 months (median, 60 months). Complete or partial biochemical and/or partial tumor response was achieved in 47.1% (responders). No significant biochemical or tumor response was seen in 23.5% and deterioration in biochemical and tumor outcomes was seen in 29.4% (non-responders). None of the patients showed complete biochemical and tumor responses. In responders, these effects were documented within 4 months after initiation of CVD with a progression-free survival of 31 to 60 months (median, 40 months); they also had improvements in hypertension and impaired glucose tolerance [83]. A meta-analysis consisting of four studies concerning a total of 50 patients with advanced/metastatic PHEO/PGL showed that a partial response could be achieved in 37% of patients [84]. Anecdotal reports suggest that the efficacy of chemotherapy may be high in patients with mutations in *SDHB* [85]. Although the CVD regimen led to an overall response of approximately 50%, it is not clear if the administration of CVD impacts overall survival, as nearly all patients develop progressive and ultimately fatal disease [82].

### Peptide receptor radionucleotide therapy (PRRT)

PHEO/PGL often expresses somatostatin receptor types 2 (SSTR2) and 3 (SSTR3) [86, 87]. Panntetreotide, DOTATOC, and DOTATATE are somatostatin analogs (SA) that target somatostatin receptors (SSTR). These analogs have been labeled with indium (^111^In), gallium (^68^Ga), yttrium (^90^Y), and lutetium (^177^Lu) and have been used extensively in the detection and therapy of a variety of neuroendocrine tumors [88]. A meta-analysis of studies involving advanced/metastatic PHEO/PGL patients treated with PRRT showed that 89.8% of pooled patients had achieved disease stabilization or a partial response [31]. Menda et al. treated 17 children with neuroendocrine tumors with ^90^Y DOTATOC [89] including 2 patients with neuroblastoma and three with PGL. Doses ranged from 30 to 50 mCi/m^2^ per cycle with intent to deliver 3 cycles. One patient received 133 mCi and had stable disease at the end of therapy. PGL patients received total doses ranging from 125 to 292 mCi. Two had stable disease and one had a minor response. All three PGLs had relief of bone pain and were alive at follow-up 17–84 months later.

Twelve patients with PGLs (with one PHEO) who received _177_Lu-DOTATATE were the subject of a report by van Essen et al. [88]. These patients received single doses of 200 mCi with intention to retreat 3 to 4 times at 6–10 weeks. The total doses administered ranged from 405 to 800 mCi. After treatment, 6 patients had stable disease, 1 had a partial response and 1 had minor response. The authors concluded that there was evidence of therapeutic effect, although it appeared lower than that in gastroenteropancreatic (GEP) neuroendocrine tumors (NETs). A retrospective study evaluating 28 PHEO/PGL patients treated with ^90^Y DOTATOC [90] included 19 patients who received 2 cycles of 100 mCi at 8-week intervals; 6 patients received 4 cycles of 50 mCi/m^2^ at 6-week intervals, and 3 patients received 1 cycle of 100 mCi/m^2^ followed by 200 mCi of ^177^Lu at 8-week intervals. There were 2 partial remissions, 5 minor responses, 2 mixed responses, and 13 patients with stable disease at restaging.

Moreover, Kong et al. reported on 20 patients with advanced/metastatic PHEO/PGL and high SSTR expression, treated with ^177^Lu-DOTA-octreotate to determine the efficacy of PRRT in controlling hypertension. At 3 months after PRRT, 8 of the 14 patients treated for HTN required reduced medication doses; 5 had no change in antihypertensives, and 1 was lost to follow-up. Thirty-six percent had disease regression (29% partial and 7% minor response) on computed tomography, with stable findings in 50%. Three other patients had bony disease evaluable only on SSTR imaging (2 partial response and 1 stable). Median progression-free survival was 39 months; median overall survival was not reached (5 deaths; median follow-up, 28 months) [91].

Furthermore, a study with 22 patients with progressive or metastatic PHEOs/PGLs were treated with PRRT, with either ^90^Y-dotatate or ^177^Lu-dotatate, or with ^131^I-MIBG treatment. Patients treated with PRRT had increased PFS and response to treatment compared with ^131^I-MIBG-treated patients (*p* < 0.05). However, there were no overall survival differences [92]. Currently, there is an ongoing trial with ^177^Lu-dotatate for inoperable PHEO/PGL (NCT03206060).

Somatostatin agonists such as lanreotide or long-acting octreotide in combination therapy and/or maintenance therapy may play a significant role in tumor control in patients with GEP-NET who are undergoing PRRT treatment [93, 94]; however, the clinical benefit in PHEO/PGL is unclear and further studies are warranted.

### Temozolomide

Temozolomide (TMZ) is a 3-methyl analogue of mitozolomide, which was developed as an orally administered alternative to intravenous dacarbazine [95] and has shown antitumor activity similar to dacarbazine in the treatment of melanoma [96]. The efficacy of TMZ for the treatment of glioblastoma and neuroendocrine tumors is correlated with the expression of O(6)-methylguanine-DNA methyltransferase (*MGMT*) and/or *MGMT* promoter methylation [75, 97].

More recently, a retrospective study showed therapeutic benefit of TMZ in patients with metastatic PGL. There was a correlation between *SDHB* mutation and O(6)-methylguanine-DNA methyltransferase (MGMT) promoter methylation and MGMT expression and response to temozolomide in the French nationwide independent cohort of 190 PHEOs or PGLs. PFS according to RECIST 1.1 and PERCIST 1.0 criteria was the primary end point. Fifteen consecutive patients with metastatic PGL were enrolled; ten (67%) carried a mutation in *SDHB*. The mean dose intensity of TMZ was 172 mg/m^2^ daily for 5 days every 28 days. Median PFS was 13.3 months after a median follow-up of 35 months. There were five partial responses (33%), seven stable (47%), and three progressive diseases (20%). The grade 3 toxicities observed were lymphopenia in two patients and hypertension in one. Partial responses were observed only in patients with a mutation in *SDHB*. MGMT immunohistochemistry was negative in tumor samples from four patients who responded to treatment. SDHB germline mutation was associated with hypermethylation of the MGMT promoter and low expression of MGMT in 190 samples of the French nationwide independent cohort [98]. This study demonstrates that TMZ is an effective antitumor agent in patients with *SDHB*-related metastatic PGL. The silencing of MGMT expression as a consequence of MGMT promoter hypermethylation in *SDHB*-mutated tumors may explain this finding.

Furthermore, an anecdotal report of a case with metastatic PGL to the liver suggested that an oral regimen of temozolomide is associated with antitumor activity. In this report, short-term treatment resulted in significant clinical improvement, a catecholamine secretory reduction rate of 75%, and normalization of abnormal hepatic function. In addition, there were minimal, but measurable, reductions in the sizes of the primary tumor and hepatic metastases. The clinical improvement and biochemical responses allowed for a surgical debulking procedure to proceed successfully without any complications [99].

Two anecdotal reports describe cases of SDHB PGL who responded to regimens of metronomic TMZ and high-dose lanreotide. One patient achieved progression-free survival for 13 months, and the second patient remained under treatment after 27 months of stable disease. Treatment was well tolerated in both cases [100]. As previously discussed, olaparib, an FDA-approved PARP inhibitor, was able to amplify the therapeutic effect of temozolomide in *SDHB-*mutant preclinical models [69]. Furthermore, the combination of PARP inhibitors and TMZ has been evaluated in other types of solid tumors. Farago et al. recently reported a phase I/II trial of combination olaparib with TMZ in patients with small cell lung cancer (SCLC). They established the recommended phase II dose of olaparib 200 mg orally twice daily in combination with temozolomide 75 mg/m^2^ daily, on days 1 to 7 of a 21-day cycle. However, further studies are warranted with this treatment modality in patients with metastatic PHEO/PGL [101].

## Conclusions

New treatments are emerging for patients with advanced/metastatic PHEO/PGL. PHEO/PGL tumors that are driven by germline mutations can be grouped into three different genetic clusters. Certain therapies may be more effective based on the underlying genetic mutation of the tumor. Although retroprospective analyses demonstrate that the CVD regimen results in an overall response of approximately 40–50%, there are multiple limitations in the data supporting this conclusion and the therapeutic potential of CVD. These limitations include the small size of the studies, the retrospective analyses, the absence of impact on overall survival, and the associated toxicity profile. Although the FDA approval of Azedra® provides a therapeutic alternative, the accessibility to this treatment modality remains limited. Given these limitations, new therapeutic approaches are necessary to improve overall survival and quality of life in patients with advanced/metastatic PHEO/PGL.
